# Millimeter wave photonics with terahertz semiconductor lasers

**DOI:** 10.1038/s41467-021-21659-6

**Published:** 2021-03-03

**Authors:** Valentino Pistore, Hanond Nong, Pierre-Baptiste Vigneron, Katia Garrasi, Sarah Houver, Lianhe Li, A. Giles Davies, Edmund H. Linfield, Jerome Tignon, Juliette Mangeney, Raffaele Colombelli, Miriam S. Vitiello, Sukhdeep S. Dhillon

**Affiliations:** 1 Laboratoire de Physique de l’Ecole Normale Supérieure, ENS, Université PSL, CNRS, Sorbonne Université, Université de Paris, Paris, France; 2 Centre de Nanosciences et de Nanotechnologies (C2N), CNRS UMR 9001, Université Paris-Saclay, Palaiseau, France; 3 NEST, CNR - Istituto Nanoscienze and Scuola Normale Superiore, Piazza San Silvestro 12, Pisa, Italy; 4 DOTA, ONERA, Université Paris-Saclay, Palaiseau, France; 5 School of Electronic and Electrical Engineering, University of Leeds, Leeds, UK

**Keywords:** Microwave photonics, Quantum cascade lasers, Nonlinear optics, Terahertz optics

## Abstract

Millimeter wave (mmWave) generation using photonic techniques has so far been limited to the use of near-infrared lasers that are down-converted to the mmWave region. However, such methodologies do not currently benefit from a monolithic architecture and suffer from the quantum defect i.e. the difference in photon energies between the near-infrared and mmWave region, which can ultimately limit the conversion efficiency. Miniaturized terahertz (THz) quantum cascade lasers (QCLs) have inherent advantages in this respect: their low energy photons, ultrafast gain relaxation and high nonlinearities open up the possibility of innovatively integrating both laser action and mmWave generation in a single device. Here, we demonstrate intracavity mmWave generation within THz QCLs over the unprecedented range of 25 GHz to 500 GHz. Through ultrafast time resolved techniques, we highlight the importance of modal phases and that the process is a result of a giant second-order nonlinearity combined with a phase matched process between the THz and mmWave emission. Importantly, this work opens up the possibility of compact, low noise mmWave generation using modelocked THz frequency combs.

## Abstract (web summary)

Photonic solutions for generating free space millimeter radiation is a fast developing field that combines optoelectronics and RF domains but has many challenges. Here the authors present a quantum cascade laser (QCL) based solution for THz laser emission and millimeter wave generation in a single device.

## Introduction

The ability to generate millimetre (mmWave) waves (30–300 GHz) from highly stable lasers promises to be a key method to achieve the spectrally pure, low phase noise and microwave signals that are targeted for local oscillators in high frequency communications^1–3^. Examples have been shown by heterodyning two optical lasers^4^, opto-electronic oscillators^5^ and pulsed lasers^6^, in a nonlinear medium or a fast photodetector to generate microwaves signals. This field of microwave photonics has also been revolutionised by the advent of the optical frequency comb, where the beating of the highly stable modes can generate low noise and high purity microwave emission^7–9^. Most of these methods, however, use lasers operating in the visible or near-infra-red part of the electromagnetic spectrum, leading to orders of magnitude difference in photon energy between the generating laser photon energy and that of the microwave photon – the quantum defect. This inherently limits the efficiency of the system ~ *E*_GHz_*/E*_opt_, where *E*_opt_ is typically 1 eV and *E*_GHz_ < 1 meV. Finally, microwave photonic systems based on optical frequency combs are typically used in a non-integrated approach, where the optical laser is separated from the fast photo-mixer or nonlinear crystal^7,9,10^. Although a range of electronic methods can be used to generate directly or through upconversion mmWave emission^11–13^, these sources typically suffer from high phase noise and therefore not as spectrally pure as photonic based sources^7,14^. In this work we show that Terahertz (THz) frequency quantum cascade lasers (QCLs)^15^, with their inherently low photon energies, two orders of magnitude smaller than in the optical range, can be used as an integrated platform to generate free space emission over the entire mmWave region (0.02–500 GHz) via intracavity nonlinear beating of the laser longitudinal modes.

THz QCLs operate between 1.2 and 5 THz, corresponding to photon energies of 5–21 meV, and possess ultrafast dynamics and giant nonlinearities^16^ permitting an integrated approach where the laser excitation and mmWave generation are realised within the same laser cavity. THz QCLs have achieved impressive performances in term of spectral coverage^17^, output power^18^ and bandwidth^19,20^ via carefully designed waveguides and active regions. Further, temperature operation has been extended to Peltier cooled operation^21,22^, with recent results showing operation up to 250 K^23^ with further perspectives to achieve room temperature operation through new bandstructure designs and materials^24^. To extend the capabilities of these devices, nonlinear intracavity processes have been successfully exploited to generate frequencies in the telecom band ~1.55 µm^25,26^, and shown the generation of frequency combs^27–29^ with octave spanning bandwidth^20^. Furthermore, difference-frequency generation (DFG) in mid-infrared QCLs has shown the possibility of efficient THz generation at room temperature^30–33^. These demonstrations illustrate the giant optical nonlinearities that can occur within QCLs (greater than ~10^5^ pm/V, compared to 10^2^ pm/V for GaAs) and how they can be used to access different spectral regions. However, the possibility of accessing the entire mmWave region, either through direct or nonlinear generation, has yet to be demonstrated. Recently, mid-infrared QCL-based frequency combs were shown to generate free space microwave radiation through beating of the comb lines in an integrated approach^34,35^. This takes advantage of the fast QCL dynamics (~1 ps), suggesting potential operation even up to THz frequencies, and highlighted both electronic and photonic methods to generate electromagnetic radiation within QCLs. However, such microwave emission has, so far, been limited to low frequencies with optical beatnote detection up to 13 GHz shown on a Schottky mixer^36^. This results in a large spectral gap between 15 and 500 GHz for QCL-based microwave generation.

Here we demonstrate free space mmWave generation in a set of THz QCLs with different dimensions, emission bandwidth and spectral response. As a common characteristic, all devices show mmWave emission lines, equally spaced in frequency by the QCL round-trip frequency. Depending on the bandwidth of the THz QCL employed and the dispersion of the THz lines, we realise mmWave emission up to 500 GHz. Moreover, we demonstrate that the frequency of the mmWave lines can be engineered by designing a QCL with two-separated THz spectral bands, with the formation of the mmWave emission measured on ultrafast timescales. We also highlight the important role of modal phases for DFG and we show here that the mmWave generation is a result of a phase matched process, owing to low loss waveguides and to the phase of the mmWave emission being equal to the envelope of the THz emission. Finally, we show a giant second-order nonlinearity from the QCL bandstructure, owing to the closely spaced sublevels in energy that directly permits a nonlinearity in the mmWave range. This could support the stabilisation of QCL-based frequency combs, in addition to four-wave mixing.

## Results

To characterise the entire QCL emission from 10 to 4000 GHz, we employ an injection seeding technique based around electro-optic detection in a THz-TDS system. As well as enabling ultrafast time-resolved measurements, this proven technique provides a detection bandwidth much greater than spectrum analysers and limited only by the cut-off frequency of the ZnTe electro-optic detection crystal^37^. To demonstrate mmWave generation, a coherent terahertz pulse generated from an ultrafast laser is injected into the QCL, and at the same time it is switched on with an ultrafast electrical pulse that is synchronised to the THz pulse. The pulse is amplified to the steady state where it seeds the QCL emission. This permits free space coherent detection of the QCL time-resolved electric field using a second beam from the same ultrafast laser^38^. The technique has shown to be equivalent to laser action on the inherent QCL spontaneous emission such that the measured time profile corresponds to the free-running case^39^. A squared Fourier transform operation is applied to the measured time-domain trace in order to extract the QCL intensity spectrum. In this study, three QCLs with lasing frequencies between 2 and 3 THz with metal-metal waveguides^40,41^ were used. For QCL_1_ and QCL_3_, the active region design is based on a 2.4-THz three well^42,43^ structure, whilst QCL_2_ is based on a 2.8-THz heterogeneous stacked nine well design^29,44,45^ for broadband operation (~800 GHz). QCL_1_ shows laser action with a bandwidth of ~500 GHz and is 1.5 mm long and 60 µm wide. QCL_2_ is 2.9 mm long and 85 µm wide. Finally, QCL_3_ was designed for two lasing bands separated by ~240 GHz using an integrated Gires-Tournois interferometer^46^ (GTI) and is 3 mm long and 68 µm wide. The GTI is 60 µm long, and separated from the main QCL section by 4 µm. Each QCL is used to show a range of spectral responses in the mmWave generation as discussed below.

**Fig. 1:**
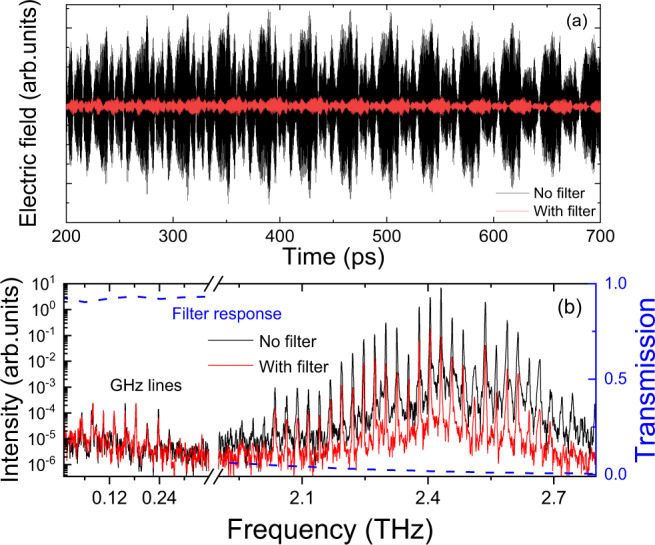
Temporal and spectral response of mmWave generation from QCL_1_. **a** Time-resolved electric field from injection seeded QCL with (red) and without (black) the low-pass filter. Electric field oscillations of the laser emission are not visible owing to the long temporal scan. **b** Intensity spectrum of emission from QCL_1_ with (red) and without (black) the low pass filter. The QCL is driven in pulsed mode (10% duty cycle) and at a fixed heat sink temperature of 10 K. The THz emission is centred around 2.4 THz. mmWave generation is clearly observed. The filter (blue curve) removes an order of magnitude of the intensity of the THz emission but does not change the mmWave emission. This unambiguously illustrates the mmWave emission is from the QCL and not an artefact of the detection technique.

**Fig. 2:**
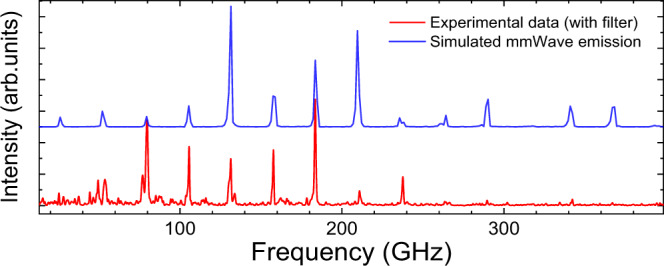
Enhanced view of mmWave spectral emission from QCL_1_ (red) showing comparison with simulated results (blue) from the QCL THz spectrum. mmWave emission is observed up to 367 GHz.

The emission properties of QCL_1_ are shown in Fig. 1. The temporal response (Fig. 1a) shows the typical profile from these devices^38,43^ with a strong amplitude modulation that can arise from the QCL ultrafast dynamics^39^. The corresponding frequency spectrum (Fig. 1b) shows emission centred at 2.42 THz with Fabry-Pérot modes separated by the round-trip frequency (*f*_*RT*_). Owing to the broadband nature of the coherent detection, the spectrum also clearly shows spectral modes covering the mmWave region between 26 GHz and 367 GHz, also separated by exactly *f*_*RT*_. In order to rule out any spectral artefact originating from nonlinearities in the measurement arrangement that are not associated with the QCL, we additionally conducted the investigations employing a 500-GHz low-pass filter (Spectrasil B). The transmission spectrum is shown in Fig. 1b (blue dotted curve). Although the THz signal intensity is attenuated by more than one order of magnitude, the mmWave lines remain the same, demonstrating that the emission originates from the QCL. An expanded view of the mmWave spectrum is shown in Fig. 2. Two observations can be made. First, there is an equal mode spacing between the THz and the mmWave lines (~ 26 GHz), with the latter’s bandwidth limited to the bandwidth of the THz emission. Second, the position and intensity of the mmWave lines can be evaluated by calculating the expected positions from the measured THz temporal emission whilst assuming a second-order nonlinearity for DFG. This is done by calculating the Fourier transform of the square of the THz temporal emission (blue curve in Fig. 2), as the second-order polarisation, *P*^2nd^, is proportional to the square of the electric field, *E(t)*. This allows the phase of each mode to be automatically considered in the calculated emission spectrum. The effect of the phase is discussed further below in the case of QCL_2_. The collection and detection efficiencies are included in the calculation and were estimated by 3D simulations of the far-field (COMSOL Multiphysics) and the waist size of each frequency at the detection crystal, respectively. These factors lead to reduced detection response to low-frequency mmWave emission and further details are highlighted in the supplementary material Figs. 2 and 3. Figure 2 shows an excellent coincidence with the frequencies of the mmWave and reasonable agreement with intensities of the mmWave modes with the lowest modes showing weak emission. Indeed, the fundamental mode at ~ 26 GHz is barely observed, although the second harmonic is weaker than the third, in contrast to the simulations. The detection of the fundamental electrical beatnote is discussed further below.

**Fig. 3:**
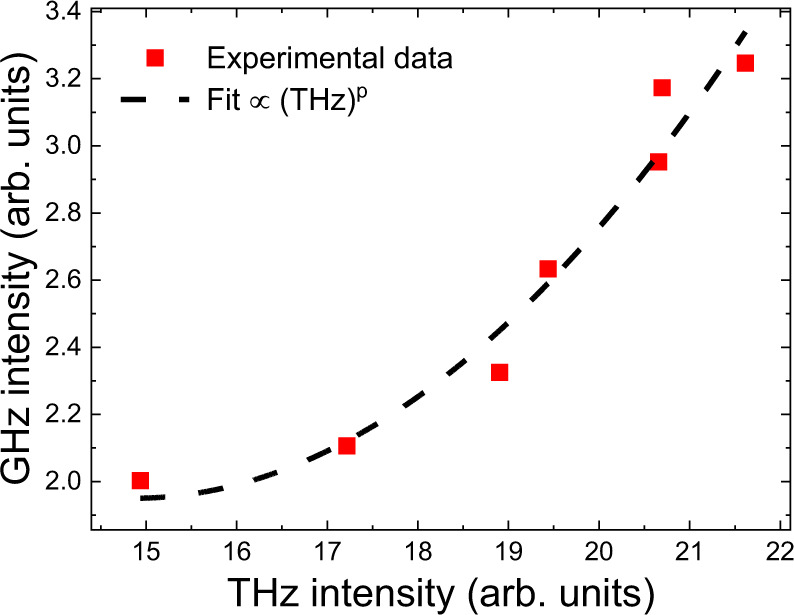
Observation of second-order nonlinearity. mmWave intensity as a function of THz intensity (red squares) illustrating a nonlinear behaviour with an exponent of *P* = 2.1 (fit, black dashed line) corresponding to a second-order susceptibility. The measured intensities correspond to the integrated emission in each spectral band.

As well as simulating the expected spectrum, a power dependence measurement was performed to further show that the mechanism is based on a second-order nonlinearity. From the spectrum acquired from the temporal trace, the integral of the mmWave spectrum as a function of the integrated THz intensity was measured, with the THz intensity varied with the QCL bias (see Fig. 3). A power law is used to fit the experimental data and the best agreement is found for a value of the exponent 2.10 ± 0.33. This identifies the second-order nonlinear susceptibility, χ^(2)^, as the main contribution to the nonlinear optical process. The small deviation from an exponent equal to 2 is most likely a result of not efficiently detecting the low harmonics. To conclude, QCL_1_ shows equally spaced mmWave lines from 25 to 367 GHz with an agreement between the mode intensity distribution of the measured and the calculated GHz lines through a second-order nonlinearity.

**Fig. 4:**
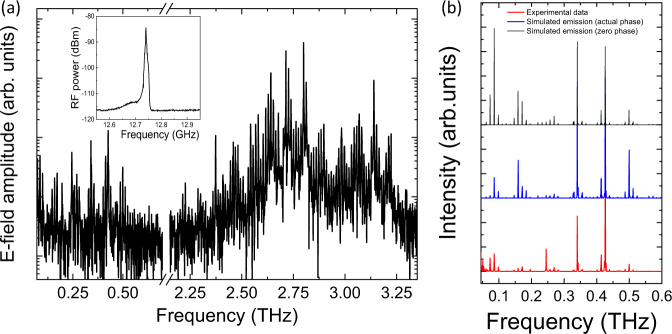
mmWave generation from broadband QCL (QCL_2_). **a** Full amplitude spectrum showing THz emission centred at 2.8 THz with a bandwidth of 800 GHz and nonlinear mmWave generation up to 500 GHz. Inset - fundamental electrical beatnote from THz QCL detected using the QCL as a detector and a spectrum analyser. **b** Enhanced view of mmWave intensity spectrum (red) compared to simulated emission for THz spectrum with measured phases (blue) and assuming equal phases (black). Spectra offset for clarity. The QCL is driven in pulsed mode (10% duty cycle) and at a fixed heat sink temperature of 10 K.

With the proof of principle of mmWave DFG demonstrated, QCL_2_, designed for ultra broadband THz emission, showed mmWave generation with an increased bandwidth extending to 500 GHz (see Fig. 4a). However, owing to the irregular power distribution between the THz QCL Fabry-Pérot modes, the mmWave modal distribution is also irregular, in agreement with the simulations shown in Fig. 4b (blue curve), calculated as described above. Here, the expected spectrum is further compared to the case where the phases of the modes are assumed to be equal (black curve). Although the frequencies are the same, the modal intensities at low frequencies (<100 GHz) are considerably higher than those measured. This highlights strongly the effect of the modal phases on the difference-frequency generation process in the case of neighbouring Fabry-Pérot modes. Furthermore, similar to observations in QCL_1_, QCL_2_ demonstrated that the mmWave modes at low frequencies were weak compared to that expected, with the fundamental mode missing. However, as QCL_2_ was 3 mm long, the electrical and free-space fundamental beatnote is expected to be around 13 GHz. This was then electrically detected^36^, using the QCL itself as an ultrafast detector and a standard spectral analyser (inset Fig. 4a), despite it being missing in the free space spectrum. This supports the above simulations for QCL_1_ that the free space optical modes at lower frequencies are not efficiently detected. This is a result of the strong diffraction owing to the subwavelength dimensions of the QCL waveguide, large focused spot sizes at these frequencies, combined with THz optics (parabolic mirrors) that have a limited collection efficiency. The detection of the narrow electrical beatnote also suggests a coherence between the Fabry-Pérot modes, which can be potentially locked to an external reference for modelocking. The high frequency microwave signals would also possess similar coherence properties.

**Fig. 5:**
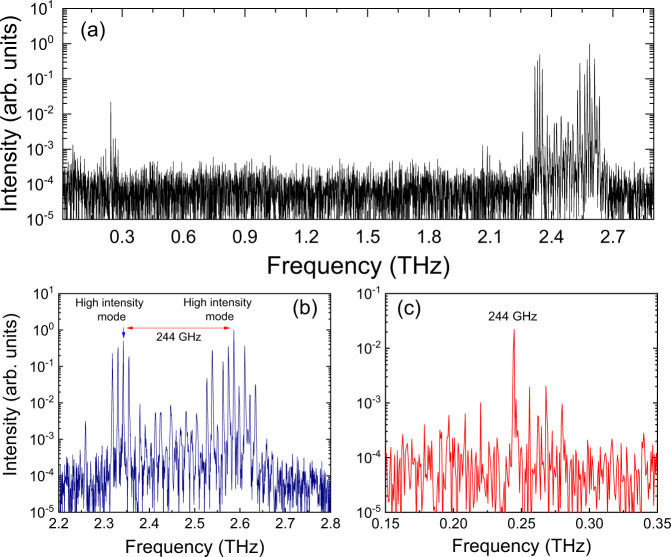
mmWave emission engineering from QCL_3_. **a** Full spectrum showing THz emission split in two spectral bands around 2.45 THz using an integrated GTI. Nonlinear mmWave generation centred at 244 GHz is observed. **b** Enhanced view of THz emission showing the two spectral bands with mode separation of 14 GHz. **c** Enhanced view of nonlinear mmWave emission showing emission centred at 244 GHz. The QCL is driven in pulsed mode (10% duty cycle) and at a fixed heat sink temperature of 10 K.

**Fig. 6:**
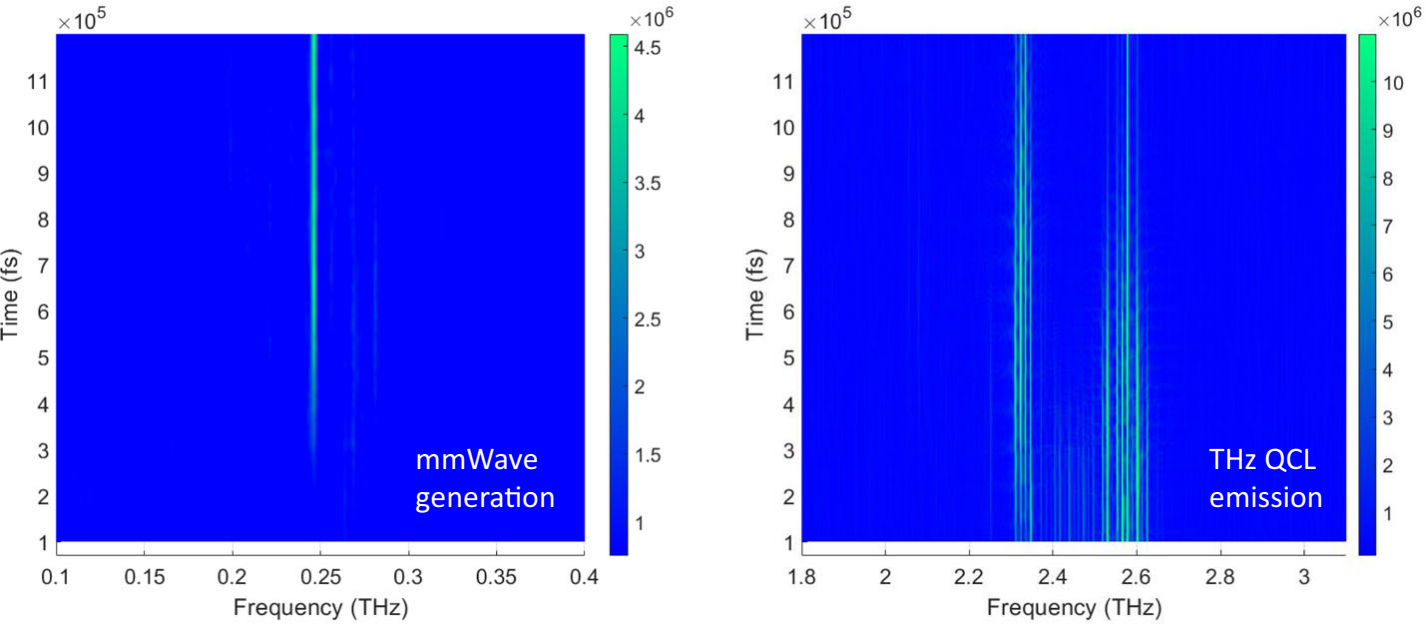
Time resolved spectral emission from QCL_3_. The intensities of the spectral modes plotted as a function of frequency (*x*) and time in femtoseconds (*y*-axis), showing the build-up and seeding of the THz QCL emission at ~400 ps centred ~2.45 THz (right) followed by the generation of stable mmWave emission at 244 GHz (left). The intensity of the emission (arb. units) is shown by the colour bar on the right of each figure.

For the final sample, QCL_3_, we show how the mmWave emission can be controlled to emit predominately at a single frequency by engineering the THz QCL spectral emission using dispersion management. Research using MIR QCLs^30^ has exploited imbedded gratings to force the QCL to emit at two distinct spectral lines for THz generation by DFG. Here we designed a THz QCL with an integrated GTI^46^ that permits the QCL to lase on distinct and separated spectral bands. Where previous work was based on placing the GTI off-resonance to reduce the total dispersion, here the centre of the QCL emission is placed in a dispersive regime that permits the generation of the spectral bands. The same active region as in QCL_1_ was used with a GTI of length 60 µm. The full emission spectrum of QCL_3_ is shown in Fig. 5a showing both the mmWave DFG and the THz emission. Figure 5b shows an expanded view of the THz spectra, where the THz QCL emits in two THz spectral bands, each spanning several tens of gigahertz. The highest intensity modes are at 2.586 THz and 2.342 THz with a frequency separation of 0.244 THz. An expanded view of the mmWave spectra is shown in Fig. 5c where the spectral position of the mmWave signal is in exact agreement with the difference in the frequencies of the main THz peaks. The intensity of the mmWave emission is equal to 0.02 percent of the highest pump intensity at 2.586 THz. This demonstrates the possibility of tailoring the desired mmWave spectrum by carefully engineering the THz emission, opening up the possibility of extending the emission towards 1 THz using octave spanning QCLs. With QCL_3_ showing strong emission at mmWave frequencies, the time evolution of the spectrum was studied and shown in Fig. 6 as a colour plot. The intensities of the modes are plotted as a function of frequency (*x*) and time in femtoseconds (*y*-axis). This shows clearly that the mmWave emission (left curve) only takes hold after the QCL emission (right curve) is established and stable, here from ~400 ps. Figure 6 also shows the interesting dynamics of the system where before 400 ps corresponds to the initial transient stage where all modes experience some gain and hence amplified over a few round trips. Here it can be seen that the modes in-between the two main parts of the spectrum experience less gain owing to the GTI reflectivity. After 400 ps the laser reaches a steady-state region corresponding to laser action and where the cavity (GTI+laser) establishes the spectral emission. This behaviour is similar to the dynamics observed in DFB THz QCLs^47^, which was compared to a reduced rate formulism.

## Discussion

**Fig. 7:**
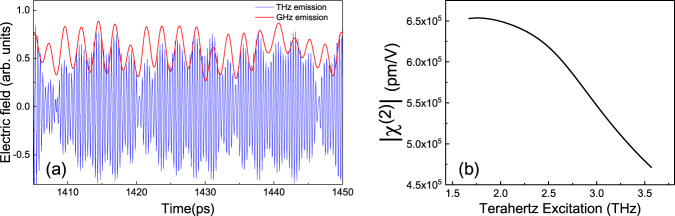
Phase matching and nonlinear susceptibility between mmWave and THz emission. **a** Electric field as a function of time for both mmWave (red curve, filtered response of THz spectrum) and THz emission (blue) over a short time window illustrating the phase matched process between the two emission bands, **b** Modulus of second-order nonlinear susceptibility χ^(2)^ as a function of THz excitation energy, calculated from the QCL bandstructure, showing the exalted giant nonlinearity.

Finally, the efficiency of the process can be used to determine the origin and the nonlinear susceptibility arising from difference-frequency generation^25,30^. The efficiency of QCL_1_, defined here as the ratio between the integrated mmWave and the THz spectrum, was found to be ~1.7 × 10^−4^. Previously work has indicated the possibility of phase matching in measurements of the microwave transmission^48^ and pulse generation in THz QCLs^43,49^. Here, using the time resolved nature of our experimental technique, we show directly the process is phase matched between the THz QCL emission and the nonlinear mmWave generation. Figure 7a shows the electric field of the THz QCL emission and the mmWave emission in the seeded regime. It is clear that the mmWave emission is in phase with the envelope of the THz emission, i.e., the phase velocity of the former is equal to the group velocity of the latter. This is a result of the low dispersion between these spectral ranges compared to the mid- and near-infra-red and the relatively low mmWave losses in these microstrip-type waveguides. Taking this phase matched process, from the efficiency a second-order susceptibility χ^(2)^ ~ 1 × 10^5^ pm/V over the mmWave region is estimated with a THz intracavity power of ~20 mW. This giant nonlinearity when compared to GaAs (~100 pm/V) is comparable to other intersubband systems and is a result of the large dipoles between closely spaced subbands^32^. Indeed the calculated value from the QCL bandstructure^30^ is χ^(2)^ ~ 6 × 10^5^ pm/V, highlighting that the nonlinearity is a result of resonant excitations of the subbands (see Fig. 7b and supplementary material Fig. 4 for details on the simulation). The measured susceptibility is lower and could be a result of the lack of sensitivity of the detection scheme to low microwave frequencies as discussed above. Further, all the carrier population is assumed to be in the upper laser that tends to overestimate the nonlinearity. By engineering the dipole and nonlinear interferences between subbands^50^, further perspectives to engineer the response of the mmWave emission for higher efficiencies are possible.

To conclude, we demonstrate the generation of narrowband free space mmWave radiation that can cover the entire 26–500 GHz range. This is based on the beating of the Fabry-Pérot modes of THz QCLs and using electro-optic detection for sensitivity over the full microwave, mmWave and THz regions. A range of functionalities were shown with regular mode spacing up to 367 GHz, mmWaves generated up to 500 GHz using broadband QCLs and engineering of the mmWave emission by using integrated GTIs. The generation process was shown to rely on a second order process and thus indicates a second-order nonlinearity. The down-conversion of THz frequency lines into microwave and sub-THz emission is also a promising process to achieve continuous emission from a few GHz to several THz using multi-octave spanning QCLs. Further, owing to the demonstrated frequency comb nature of these sources and the increasing temperature operation of THz QCLs, the generation of extremely low-noise mmWave emission at hundreds of GHz is feasible. This would potentially permit the application of these sources as local oscillators for future high-frequency free space telecommunications.

## Methods

### Experiment

The temporal characterisation of the THz quantum cascade laser (QCL) is based on coherent sampling of the electric field (E-field) using electro-optic detection. This technique requires the emission of the THz QCL to be phase locked to a THz pulse, and thus locked to the repetition rate of a femtosecond laser. An established ultrafast injection seeding technique is employed to realise this condition. A broadband THz seed pulse with a fixed phase is generated using an interdigitated photoconductive switch excited by a femtosecond Ti:Sapphire laser (see supplementary fig. 1)^51^. The THz pulse is injected into one end of the QCL cavity as an electrical radio frequency (RF) pulse (duration of a few nanoseconds), synchronised with the seed pulse, is used to gain switch the QCL. This permits the THz input pulse to be amplified, eventually seeding the QCL emission, avoiding laser action on the QCL’s inherent spontaneous emission. The QCL emission is then measured using electro-optic sampling using a ZnTe crystal and a second beam from the same Ti:Sapphire laser.

## Supplementary information

Supplementary Information

## Data Availability

The datasets generated and analysed during the current study are available in the zenodo repository, 10.5281/zenodo.4384637
