# Supplementary material for: Millimeter wave photonics with terahertz semiconductor lasers
Source: Nat Commun. 2021 Mar 3;12:1427. doi: 10.1038/s41467-021-21659-6 (PMC7930181; doi:10.1038/s41467-021-21659-6)
Supplement: Supplementary file 1 — Supplementary Information [file 41467_2021_21659_MOESM1_ESM.pdf]

Supplementary material:
Monolithic Millimeter Wave Photonics with Terahertz Semiconductor Lasers

Valentino Pistore,[1] Hanond Nong,[1] Pierre-Baptiste Vigneron,[2] Katia Garrasi,[3] Sarah Houver,[4] Lianhe Li,[5] A. Giles Davies,[5] Edmund H. Linfield,[5] Jerome Tignon,[1] Juliette Mangeney,[1] Raffaele Colombelli,[2] Miriam S. Vitiello,[3] and Sukhdeep S. Dhillon[1*]

[1]Laboratoire de Physique de l'Ecole Normale Supérieure, ENS, Université PSL, CNRS, Sorbonne Université, Université de Paris, Paris, France

[2] Centre de Nanosciences et de Nanotechnologies, CNRS, Univ. Paris-Sud, Université Paris-Saclay, C2N-Orsay, 91405 Orsay Cedex, France

[3] NEST, CNR - Istituto Nanoscienze and Scuola Normale Superiore, Piazza San Silvestro 12, 56127, Pisa, Italy

[4] DOTA, ONERA, Université Paris-Saclay, F-91123 Palaiseau - France

[5] School of Electronic and Electrical Engineering, University of Leeds, Leeds LS2 9JT, UK

* sukhdeep.dhillon@ens.fr

Supplementary Note 1 - Injection seeding and THz time domain spectoscopy

Injection seeding: The pulse characterization of the THz quantum cascade laser (QCL) is based on coherent sampling of the electric-field (E-field) using electro-optic detection. This technique requires to phase lock the emission of the THz QCL to a THz pulse, which in turn is locked to the repetition rate of a femtosecond laser. To fulfil this requirement, an established ultrafast injection seeding technique is employed.[1] A broad-band THz pulse (seed) with a fixed phase is generated using a photoconductive switch excited by a 100 fs near-infrared pulse from a Ti: Sapphire laser (Supplementary figure 1). The THz seed pulse is injected into one end cavity of the QCL waveguide prior to gain switching the QCL with an electrical radio frequency (RF) pulse with a duration of a few nanoseconds. This allows the THz input pulse to be amplified and eventually seed the QCL emission, instead of being initiated by the QCL's inherent spontaneous emission. Finally, a purge box with dry air is used to prevent absorption of the THz emission by atmospheric water.

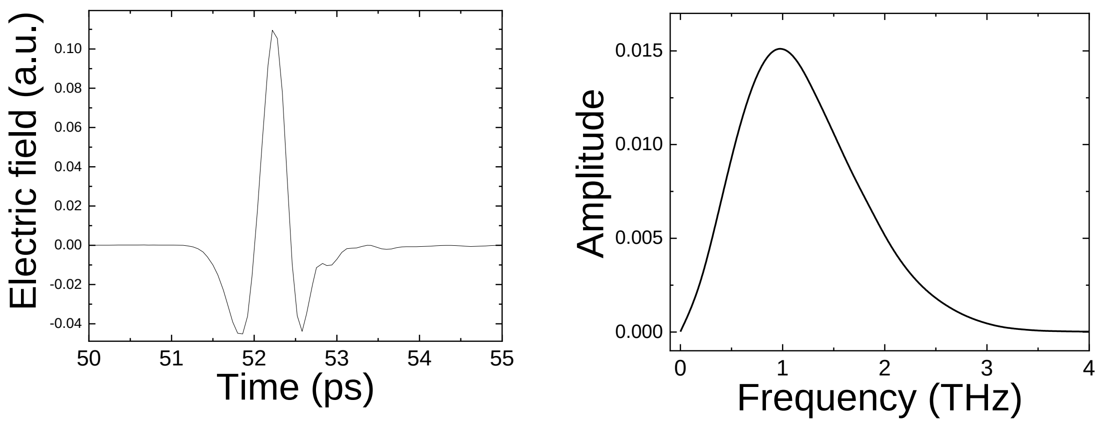

Supplementary figure 1. THz pulse characteristics. Measured Time domain (left) and Spectrum (right) of a THz pulse generated from a GaAs photoconductive antenna, measured with a ZnTe crystal using electro-optic sampling.

Supplementary Note 2 - Far field

The far-field of the mmWaves emission from the THz QCL was simulated in 3D on a Comsol Multiphysics environment. The QCL is realized as plain GaAs between two 0.1 µm thick layers of gold. The substrate was included in the simulation and the whole structure is surrounded by a sphere of air with a radius of 12.4 mm. The outer 2.4 mm thick layer of the sphere is set as a perfectly matched layer to provide a suitable absorbing boundary condition. The far-field is computed from the inner surface of this layer.

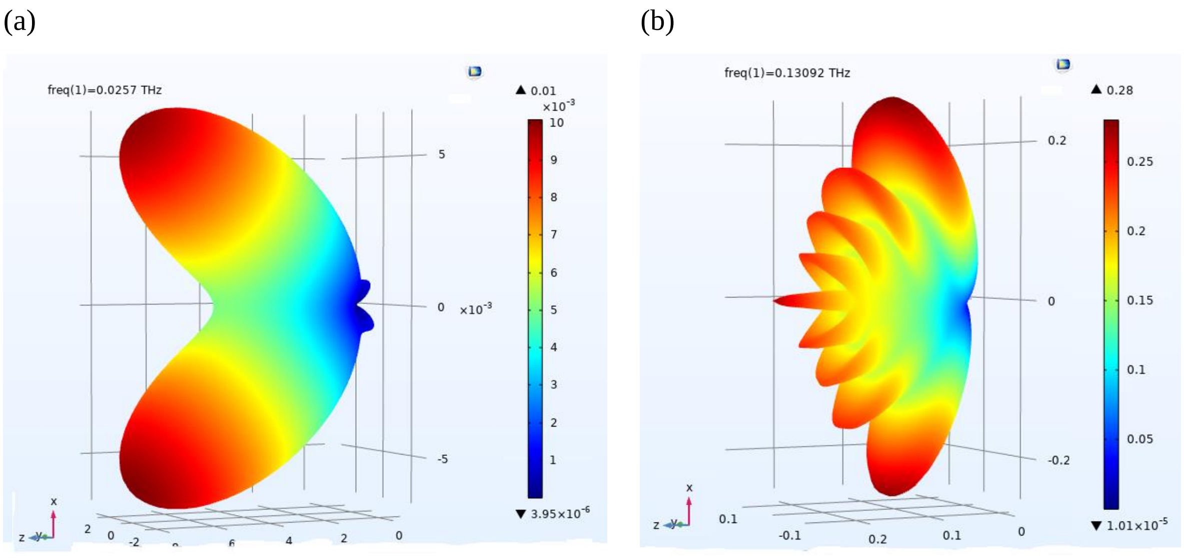

Supplementary figure 2: Far field distribution for different emission frequencies. (a) 3D plot of the power distribution in the far-field of the QCL at a frequency of 25.7GHz, corresponding to the microwave line at lowest frequency detected by the THz TDS experiments. The radiation propagates along the z axis. The two lobes are a result of the strong diffraction taking place at the facet of the device as a consequence of the strongly subwavelength confinement the radiation undergoes in the QCL cavity. (b) 3D plot of the far-field of the emission of the QCL at a frequency of 130.92GHz. The radiation is much better distributed in the z axis direction, improving the collection from the parabolic mirrors.

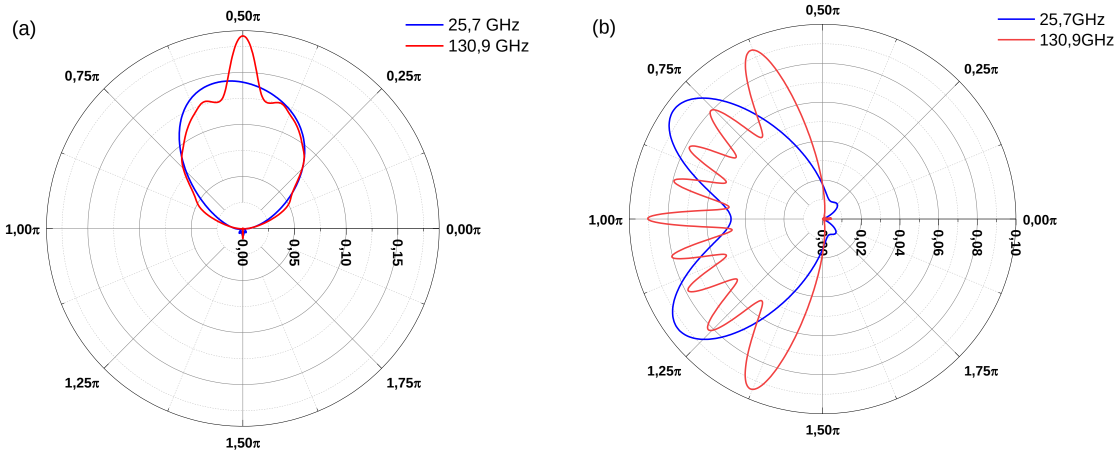

Supplementary figure 3: **Polar plots of power distribution.** (a) polar plot showing the normalized power distribution in the far-field of QCL on the xy plane (referring to the axis of supplementary figure 1) for the microwave lines at 25.7 GHz and 130.92 GHz. (b) polar plot showing the far-field of the emission of the device on the xz plane for the same frequencies of (a). Microwaves of higher frequencies are better collected by the parabolic mirror (f number = 2), corresponding to higher collection efficiencies.

Supplementary figure 2 shows two examples (at 25.7 GHz and 130.92 GHz) of the simulated 3D far-field power distribution for QCL_1. The two profiles are compared in the polar plots of supplementary figure 3. These show how the collection efficiency improves with increasing frequency of the microwave lines. In particular, microwave emission at the lowest frequencies are poorly collected by the parabolic mirrors as a result of the strong diffraction they undergo at the QCL's facets.

Supplementary Note 3 - Nonlinear efficiency

The efficiency of the non-linear process was computed from the experimental data as $\eta(\omega) = \int_{GHz} s^2_{GHz}(\omega) d\omega_1 / \int_{THz} s^2_{THz}(\omega_1)d\omega_1$ where $s_{GHz}(\omega)$ is the signal amplitude at microwave frequencies (up to 500GHz) and $\int_{THz} s^2_{THz}(\omega_1)d\omega_1$ is the integral of the THz power spectrum. The spot size of the emission at the EO crystal for each frequency is also taken into account since only the portion that overlaps with the NIR probing beam is sampled. This results in a strong underestimation of the intensity of the microwave lines at the lowest frequency. Moreover, this effect adds to the poor collection efficiency as a result of the far-field profiles at low frequencies (see above). As a result, they are less efficiently detected compared to higher frequency emission.

The non-linearity was estimated from the experimental data according to the formula:

$$\left|\chi^{(2)}(\omega)\right| = \sqrt{\frac{8\varepsilon_0 c^3 s^2(\omega) n(\omega) n(\omega_1) n(\omega_1+\omega) S_{eff}}{\int_{THz} s^2(\omega_1) s^2(\omega_1+\omega)\, d\omega_1\, \omega^2 l_{coh}^2}} \qquad [1]$$

where ε_0 is the permittivity of the free space, c is the speed of light, $n(\omega_i)$ and $s^2(\omega_i)$ are respectively the refractive index and the power at frequency ω_i, S_{eff} is the effective area of interaction and l_{coh} is the coherence length. S_{eff} and l_{coh} were assumed to be 720µm² and 1500µm respectively. The pump depletion was neglected. The term $\int_{THz} s^2(\omega_1) s^2(\omega_1+\omega)\, d\omega_1$ allows to consider each couple of points separated by ω in the power spectrum as sources for the DFG process.

Supplementary Note 4 - Nonlinear susceptibility

The QCL bandstructure and the relevant electronic levels of QCL_1 are plotted in supplementary figure 4. The laser transition, emitting photons centred at energy E_{THz}, occurs between the upper electronic level 4 (blue line) and the lower electronic level 2 (red line). To evaluate the second order nonlinear susceptibility $\chi^{(2)}$ responsible for the DFG process, we consider the difference between a fixed energy E_{THz} (here the QCL central frequency) and another laser mode frequency existing in the QCL bandwidth that act as a variable, E_{ex}. In this calculation, both E_{THz} and E_{ex} are considered monochromatic so that $\chi^{(2)}(E_{ex} - E_{THz})$ can be evaluated by Eq. 2, adapted from Ref 2. This equation calculates the overall nonlinearity resulting from the bandstructure, accounting for possible transitions between electronic levels represented in supplementary figure 4. We set N_e the electron density in the upper laser level 4, and we assume it corresponds to the doping level $N_e = 6 \times 10^{16}$ cm^{-3} (the majority of the population is expected to be in the upper laser state, as in Ref 2). n and m indexes vary from 1 to 3 corresponding to the levels below level 4 available for DFG process, z_{ij} and E_{ij} are the dipole matric element and energy

of the transition between states *i* and *j*, respectively. Γ is the broadening of the electronic transitions that we assume Γ ~ 3 meV for transitions in the THz range.

$$\chi^{(2)}(E_{ex}-E_{THz}) \approx N_e \frac{e^3}{\varepsilon_0} \sum_{m,n} \frac{z_{4m} z_{mn} z_{n4}}{E_{ex}-E_{THz}-E_{mn}+i\Gamma} \times \left(\frac{1}{E_{ex}+E_{n4}+i\Gamma} + \frac{1}{-E_{THz}-E_{n4}+i\Gamma}\right) \quad [2]$$

In order to examine different energies existing in the QCL bandwidth, we varied E_{ex} between 7 meV and 15 meV, around the QCL central frequency. The calculated modulus of $\chi^{(2)}$ is plotted as a function of E_{ex} in figure 7b of the main manuscript. Finally, we evaluate $\chi^{(2)}$ for an excitation energy around 10 meV, at the QCL central frequency, giving $\chi^{(2)} \approx 6 \times 10^5$ pm/V.

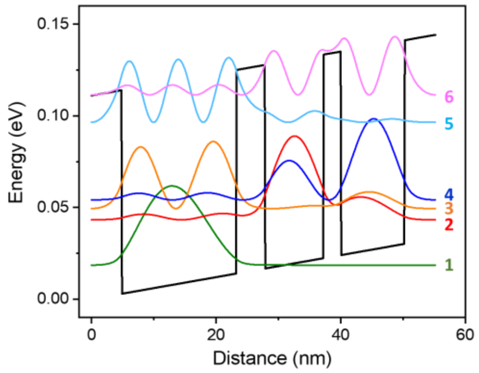

Supplementary figure 4: QCL bandstructure. Moduli squared of the relevant wave functions, shown with corresponding energies for electronic levels in the conduction band potential for QCL_1. The laser transition, emitting photons of energy E_{THz}, occurs between the upper electronic level 4 (blue line) and the lower electronic level 2 (red line).

Supplementary References

1. Oustinov, D. *et al.* Phase seeding of a terahertz quantum cascade laser. *Nat. Commun.* **1**, 1–6 (2010).
2. Belkin, M. A. *et al.* Terahertz quantum-cascade-laser source based on intracavity difference-frequency generation. *Nat. Photonics* **1**, 288–292 (2007).
